# Low hemoglobin levels are associated with Bowman’s capsule rupture and peritubular capillaritis in ANCA-associated renal vasculitis: a link of vascular injury to anemia?

**DOI:** 10.1007/s40620-023-01748-z

**Published:** 2023-09-07

**Authors:** Eva Baier, Ingmar Alexander Kluge, Samy Hakroush, Björn Tampe

**Affiliations:** 1 Department of Nephrology and Rheumatology, University Medical Center Göttingen, Georg August University, Göttingen, Germany; 2 Institute of Pathology, University Medical Center Göttingen, Göttingen, Germany; 3 SYNLAB Pathology Hannover, SYNLAB Holding Germany, Augsburg, Germany

**Keywords:** ANCA-associated renal vasculitis, Renal anemia, Peritubular capillaritis, Bowman’s capsule rupture

## Abstract

**Background:**

Anemia in anti-neutrophil cytoplasmic antibody (ANCA)-associated renal vasculitis is a severe complication that predicts renal survival. We here conducted correlative analyses to evaluate correlations of low hemoglobin levels and histopathological characteristics in ANCA-associated renal vasculitis.

**Methods:**

Fifty-two patients with biopsy-proven ANCA-associated renal vasculitis observed between 2015 and 2020 were retrospectively evaluated. Spearman’s correlation was performed to assess correlations, and statistical evaluation was performed by simple and stepwise multivariable regression.

**Results:**

Regarding laboratory anemia parameters, no significant association with serum hemoglobin levels was observed. Serum hemoglobin levels were associated with the estimated glomerular filtration rate in the total cohort (*β* = 0.539, *p* < 0.001), and in the MPO-ANCA subgroup (*β* = 0.679, *p* = 0.008). Among tubulointerstitial lesions, decreased serum hemoglobin levels correlated with peritubular capillaritis in the whole cohort (*β* = − 0.358, *p* = 0.013), and was suggested in the MPO-ANCA subgroup (*p* = 0.029, *r* = − 0.446). Regarding glomerular lesions, the prevalence of necrotic glomeruli significantly associated with low serum hemoglobin levels in PR3-ANCA (*β* = − 0.424, *p* = 0.028). In the total cohort, a significant correlation between decreased serum hemoglobin levels and the occurrence of diffuse Bowman’s capsule rupture was identified (*β* = − 0.374, *p* = 0.014), which was implied in the MPO-ANCA subgroup (*p* = 0.013, *r* = − 0.546; *p* = 0.0288, slope = − 16.65).

**Conclusion:**

Peritubular capillaritis and Bowman’s capsule rupture correlate with low hemoglobin levels; this may indicate that histopathological lesions are linked with inflammatory vascular injury and  relative erythropoietin deficiency in ANCA-associated renal vasculitis.

**Graphical abstract:**

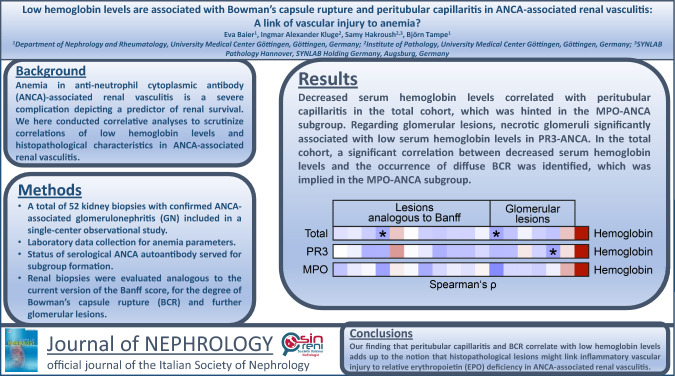

## Introduction

In anti-neutrophil cytoplasmic antibody (ANCA)-associated renal vasculitis—which is a kidney-focused term for biopsy-proven renal involvement in ANCA-associated vasculitis (AAV)—anemia is a severe complication that predicts renal survival [1, 2]. A multifactorial etiopathogenesis of anemia is generally assumed with a predominance of renal contribution, whose prevalence accounts for up to 95% [2], but pulmonary manifestations may also overlap and contribute to decreased hemoglobin levels in AAV patients, clinically presenting as granulomatosis with angiitis (GPA), microscopic polyangiitis (MPA) or eosinophilic GPA (EGPA) [3].

In general, renal anemia is a frequent complication in chronic kidney disease (CKD) patients [4]. Pathophysiologically, an erythropoietin (EPO) deficiency is the main cause, together with secondary factors such as iron deficiency, chronic inflammation and shortened life-span of red blood cells [5]. Interestingly, factual EPO deficiency is not necessarily reflected by decreased EPO levels, hence, CKD patients commonly feature EPO levels within the normal range in contrast to non-renal anemia [5]. Peritubular renal interstitial fibroblast-like cells and pericytes are mainly contributory to EPO production [6].

We recently described an association between low hemoglobin levels and need for intensive care unit supportive care in patients with ANCA-associated renal vasculitis [7]. Furthermore, levels of hemoglobin below 9.8 g/dL at admission independently predicted prolonged intensive care unit supportive care in critically ill patients with ANCA-associated renal vasculitis [7]. Kawamura et al*.* reported a correlation of tubulointerstitial damage and anemia severity, wherein a close relationship between decline of kidney function and anemia severity was repeatedly described in ANCA-associated renal vasculitis [2].

ANCA-associated renal vasculitis is a systemic autoimmune disease that affects medium- and small-sized kidney blood vessels, namely intraparenchymal arteries, arterioles, venules, glomerular capillaries, and the peritubular capillary network [8, 9]. Pauci-immune necrotizing and crescentic glomerulonephritis constitutes a histopathological hallmark, however, renal microvasculature is also affected by tubulointerstitial vasculitis that is mainly characterized by arteritis and peritubular capillaritis showing a prevalence of 26.2% and 9.5%, respectively, in one of our recent investigations [10]. In contrast to arteritis, whose implementation into the ANCA renal risk score improved end-stage kidney disease prediction [11], peritubular capillaritis is pathophysiologically relevant in the research field of kidney transplantation, where it promotes tubulointerstitial fibrosis in renal allografts [12]. Independent occurrence of peritubular capillaritis, arteritis and crescentic glomerulonephritis was implied by some case reports affirming that peritubular capillaritis causes tubulointerstitial nephritis without coinciding crescentic glomerulonephritis [13–16]. Moreover, we recently reported on Bowman’s capsule rupture (BCR) linking glomerular injury with tubulointerstitial inflammation by promoting intrarenal immune cell infiltration in ANCA-associated renal vasculitis [17, 18]. Representing an additional mode of vascular injury resulting in tubulointerstitial damage, we showed that different types of immune cell infiltrates correlate with the extent of BCR in distinct forms of ANCA-associated renal vasculitis [17].

Despite growing insight into the interplay between microvascular inflammation and tubulointerstitial damage, histopathological correlates of anemia remain elusive. Therefore, we aimed to systematically assess histopathological lesions in association with low hemoglobin levels in biopsy-proven ANCA-associated renal vasculitis.

## Methods

### Study population and inclusion and exclusion criteria

Between 2015 and 2020, 53 patients with biopsy-proven ANCA-associated renal vasculitis at the University Medical Center Göttingen (UMG), Göttingen, Germany, were evaluated and 52 were included in the present study. This patient cohort has been described in previous reports [19–24]. Inclusion criteria were: hospitalized patients aged ≥ 18 years with biopsy-proven ANCA-associated renal vasculitis including positivity for either PR3- or MPO-ANCA, available hemoglobin levels at renal biopsy, and provision of signed written informed consent. All inclusion criteria had to be fulfilled for enrollment. Exclusion criteria were all hemorrhagic events before renal biopsy affecting hemoglobin levels (including clinically manifest pulmonary hemorrhages showing hemoptysis and/or need for mechanical ventilation), whole blood transfusion before renal biopsy, double seropositivity for PR3- and MPO-ANCA, or anemia due to acute hemolysis, myelodysplastic syndrome, aplastic anemia, or other hematological disorders according to the medical records.

### Clinical data assessment and subgroup formation

Medical records were utilized to obtain data on sex, age, medication, body mass index (BMI), blood pressure levels at renal biopsy, immunosuppressive treatment shortly before or at renal biopsy (steroid pulse, cyclophosphamide [CYC], rituximab [RTX]), performance of plasma exchange (PEX) or kidney replacement therapy (KRT), and comorbidities. Acute kidney injury (AKI) was defined by AKIN classification [25]. Stages of CKD before admission were registered [26]. Laboratory data collection included blood cell counts with hemoglobin levels at renal biopsy, hemolysis parameters, peak serum creatinine levels with correlating estimated glomerular filtration rate (eGFR), blood urea nitrogen, potassium, serum complement levels C3/ C4, iron and vitamin status. The CKD-EPI (CKD epidemiology collaboration) equation was used for eGFR calculation [27]. Assessment of serum levels for MPO-ANCA and PR3-ANCA was performed using immunoassays (ImmunoCAP 250, Thermo Fisher Scientific, Waltham, USA). ANCA immunofluorescence was conducted according to the manufacturer’s protocol (EUROIMMUN AG, Lübeck, Germany). The Birmingham Vasculitis Activity Score (BVAS) was assessed as previously described [28]. Status of serological ANCA-autoantibody subtypes was applied for the formation of two subgroups: MPO-ANCA (*n* = *25*), and PR3-ANCA (*n* = *27*).

### Definitions of anemia

Anemia was defined by serum hemoglobin levels < 11.5 g/dL for female patients and < 13.0 g/dL for male patients [4]. Erythrocyte morphology was evaluated by mean corpuscular hemoglobin, mean corpuscular volume, and mean corpuscular hemoglobin concentration. Anemia was classified as renal anemia, anemia of chronic disease, anemia due to deficiency of folate, cobalamin, or iron, whereby disease overlaps were possible. Based on the clinical practice guidelines [29], renal anemia was diagnosed if an eGFR below 60 mL/min/1.73 m^2^ analogous to a CKD stage of G3 or higher for at least three months was stated in the medical records of patients, together with no evidence of exclusion criteria, folate or cobalamin deficiency. Hyporegenerative erythropoiesis (as indicated by a reticulocyte production index of less than 1.0) was evaluated, but was not considered diagnosis-defining. Moreover, coincident iron-deficiency anemia was assessed, since disease overlaps commonly occur [29]. Anemia of chronic disease was defined if decreased transferrin saturation below 16% simultaneously occurred with a normal or elevated serum ferritin concentration above 100 ng/mL, while transferrin levels within the normal range or above the upper threshold of 360 mg/dL were also assessed [2, 30, 31]. Iron deficiency anemia was confirmed when anemia occurred with low levels of transferrin saturation (< 16%) and hypoferritinemia (< 5.0 µg/L), which was registered, if no coincident elevated serum levels of C-reactive protein (CRP) prevailed [32]. Folate deficiency was diagnosed if folate serum levels were below 3.1 µg/L. Cobalamin deficiency was confirmed when serum levels below 187 ng/L occurred, while additionally serum holotranscobalamin levels below 35 pmol/L were taken into account.

### Renal histopathology

All renal biopsies were independently evaluated by two renal pathologists (IAK and SH) blinded to clinical data and analysis. Within every renal biopsy specimen, each glomerulus was scored separately for the presence of global sclerosis, necrosis, and crescentic formations. Consequently, the percentage of glomeruli with any of these features was calculated as a fraction of the total number of glomeruli in each renal biopsy. Injured glomeruli exposing crescents or necrosis were further scored for the presence of BCR, whose fractional proportionality was calculated with regard to the total amount of glomeruli with the classification of “focal” (indicating less than 50% of affected glomeruli) and “diffuse” (indicating more than 50% of affected glomeruli). Berden’s classification was assigned, as previously reported [22]. Moreover, renal biopsies were evaluated analogous to the Banff score for allograft pathology, as recently described [33, 34].

### Statistical analysis

Continuous variables were tested for normal distribution utilizing the Shapiro–Wilk test. Statistical comparisons were not formally powered or prespecified. Normally distributed variables are presented as mean ± SD, non-normally distributed variables are itemized as median (IQR), except for descriptive statistics of BCR, which is described by means of the 90% percentile. Group comparisons were performed between the PR3-ANCA and MPO-ANCA subgroups, by means of unpaired Student’s *T* test for normally distributed variables or Mann–Whitney test for non-normally distributed variables. Nonparametric between-group comparisons were performed with the Chi-Square test. Spearman’s correlation was performed to assess correlations within the total cohort and both subgroups. A Spearman’s *ρ* more than ± 0.4 in the correlation matrix was defined as relevant, and independent statistical evaluation of these parameters was performed by stepwise multivariable linear regression including variables of one predefined cluster as indicated by black rectangles in Fig. 2, italic headings in Table 2. Heatmaps represent mean values of Spearman's *ρ*. Probability values (*p* values) below 0.05 were considered statistically significant. Asterisks indicate statistically significant associations in the stepwise multivariable linear regression (*p* < *0.05*). In addition, simple linear regression was performed. Data analyses were performed with GraphPad Prism (version 9.3.1 for MacOS, GraphPad Software, San Diego, California, USA) and IBM SPSS Statistics (version 28 for MacOS, IBM Corporation, Armonk, NY, USA).

## Results

### Baseline characteristics and between-group comparisons

**Fig. 1:**
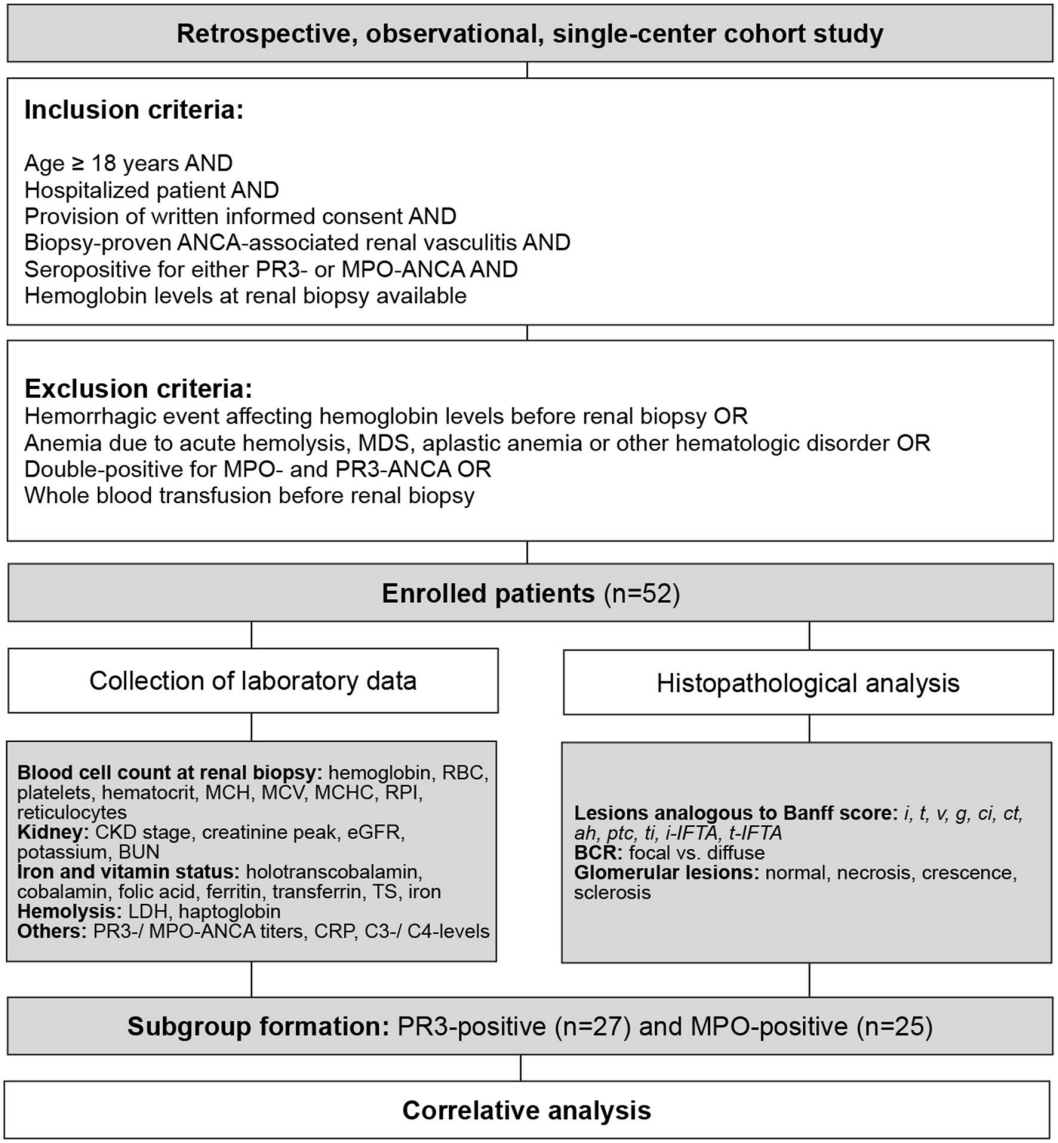
STROBE flow chart of the study. Inclusion and exclusion criteria are itemized. Abbreviations: *ah* arteriolar hyalinosis, *ANCA* anti-neutrophil cytoplasmic antibody, *BCR* Bowman’s capsule rupture, *BUN* blood urea nitrogen, *C3* complement factor C3, *C4* complement factor C4, *ci* interstitial fibrosis, *CRP* C-reactive protein, *ct* tubular atrophy, *eGFR* estimated glomerular filtration rate, *g* glomerulitis, *i* interstitial inflammation, *i-IFTA* inflammation in areas of interstitial fibrosis and tubular atrophy, *LDH* lactate dehydrogenase, *MCH* mean corpuscular hemoglobin, *MCHC* mean corpuscular hemoglobin concentration, *MCV* mean corpuscular volume, *MDS* myelodysplastic syndrome, *MPO* myeloperoxidase, *PR3* proteinase 3, *ptc* peritubular capillaritis, *RBC* red blood cells, *RPI* reticulocyte production index, *STROBE* Strengthening the Reporting of Observational Studies in Epidemiology, *t* tubulitis, *ti* total inflammation, *t-IFTA* tubulitis in areas of interstitial fibrosis and tubular atrophy, *TS* transferrin saturation, *v* intimal arteritis

Baseline characteristics are shown in Table 1. Figure 1 itemizes a STROBE flow chart of the study. Enrolled patients had a median age of 65 years, well-controlled blood pressure levels at renal biopsy, and a median hospitalization time of 16 days. Serum hemoglobin levels at renal biopsy averaged 10.2 ± 2.2 g/dL with normochromic and normocytic RBC morphology, while most enrolled patients—43/52 (82.7%) were anemic (Table 1) with no significant subgroup difference (*p* = 0.8104, Table 1). Renal anemia was observed in 9/52 cases (17.3%) also showing no subgroup difference (Table 1). Other forms of anemia are itemized in Table 1. Acute kidney injury AKIN III prevailed in 23/52 cases (44.2%) of the total cohort, while the MPO-ANCA subgroup featured more severe acute kidney damage reflected by significant subgroup differences for the prevalence of AKIN III (*p* = 0.0057, Table 1), peak creatinine levels (*p* = 0.005, Table 1) and eGFR (*p* = 0.0147, Table 1). Interestingly, the distribution pattern of CKD stages revealed no significant difference between the PR3-ANCA and MPO-ANCA subgroups (*p* = 0.3585, Table 1). A severe, chronic decrease in kidney function in terms of CKD stage G4-5 occurred in 5/52 (9.6%) patients with one case in the PR3-ANCA subgroup (1/27, 3.7%) and four cases in the MPO-ANCA subgroup (4/25, 16.0%), again displaying no significant subgroup difference (*p* = 0.1709, Table 1). Regarding the disease course, newly diagnosed cases occurred frequently in 75.0% and relapsed cases prevailed in 17.3%. Among the treatment measures, steroid pulses were by far most frequent, being administered, in 65.4% of cases compared with KRT (25.0%), PEX (21.2%), and CYC (9.6%) or RTX (7.7%, Table 1). Regarding histopathological features, between-subgroup comparisons showed significant differences in Banff-scored tubular atrophy (*ct*; *p* = 0.0347, Table 1) and significantly more normal glomeruli (*p* = 0.012, Table 1) in PR3-ANCA and total inflammation (*ti*) in the MPO-ANCA subgroup (*p* = 0.0136, Table 1). We analyzed the prevalence of anemia as per Berden’s classification and most strikingly found that all renal biopsies featuring crescentic class were accompanied by anemia (Table 1).

**Table 1:** Baseline characteristics of the total cohort, PR3- and MPO-ANCA subgroups. Group comparisons between the PR3-ANCA and MPO-ANCA subgroup

	Total (*n* = *52*)	PR3-ANCA *(n* = *27)*	MPO-ANCA *(n* = *25)*	Reference	*p* value
***Clinical data***					
Age—years	65.0 (55–65)	69.0 (55–76)	60.0 (51–70)		*0.2637*
Female sex—no. (%)	23 (44.2)	12 (44.4)	11 (44.0)		*0.9743*
BP levels at renal bx					
Systolic—mmHg	128 ± 12	127 ± 13	129 ± 11	< 160	*0.4775*
Diastolic—mmHg	74 ± 11	72 ± 13	76 ± 8	< 90	*0.2039*
BMI—kg/m^2^	24.9 (23–27)	24.4 (21–26)	25.8 (24–31)	< 25	***0.0266***
BVAS—score	18 ± 4	18 ± 4	17 ± 4		*0.3922*
Length of stay—days	16.0 (9–28)	14.0 (8–23)	19.0 (12–32)		*0.1061*
Lethal exitus—no. (%)	2 (3.8)	1 (3.7)	1 (4.0)		*0.9557*
***Blood cell count***					
Hemoglobin at rbx—g/dL	10.2 ± 2.2	10.1 ± 2.1	10.1 ± 2.2	11.5–17.5	*0.9312*
Hematocrit—%	28.7 (26–34)	28.5 (25–35)	28.9 (26–33)	39.0–51.0	*0.8523*
Red blood cells—10^6^/µL	3.4 ± 0.8	3.4 ± 0.8	3.4 ± 0.7	4.4–5.9	*0.9822*
MCV—fL	89.5 (85–93)	88.0 (86–92)	90.0 (84–93)	81.0–95.0	*0.8094*
MCH—pg	30.0 (28–31)	29.5 (29–30)	30.1 (28–31)	26.0–32.0	*0.5576*
MCHC—g/dL	33.3 ± 0.9	33.1 ± 0.9	33.5 ± 0.8	32.0–36.0	*0.1704*
Platelets—10^3^/µL	342.9 ± 186.2	408.6 ± 218.4	278.9 ± 114.0	150.0–350.0	***0.0107***
Reticulocytes—‰	17.5 (12–24)	19.5 (12–24)	17.0 (10–25)	< 25	*0.6754*
RPI—value	0.5 (0.4–0.7)	0.5 (0.3–0.8)	0.5 (0.4–0.8)	1–3	*0.9915*
***Iron status***					
Transferrin—mg/dL	146.2 ± 56.3	135.9 ± 48.3	155.1 ± 62.7	174.0–364.0	*0.3795*
Transferrin saturation—%	35.1 ± 24.1	36.3 ± 27.1	34.3 ± 22.4	14.0–48.0	*0.8365*
Ferritin—µg/L	407 (306–1452)	726.0 (350–1925)	362 (172–972)	5.0–204.0	*0.1388*
Iron—µmol/L	11.1 (5–16)	10.2 (5–13)	12.7 (6–19)	11.6–31.3	*0.2277*
Deficiency of iron—no. (%)	8/ 28 (28.6)	3/12 (25.0)	5/16 (31.3)		*0.7171*
***Vitamin status***					
Holotranscobalamin—pmol/L	64.9 (23–163)	64.9 (23–163)	–	> 35	–
Cobalamin—ng/L	397 (268–437)	424 (261–544)	361 (294–406)	187–883	*0.5338*
Folate—µg/L	5.1 ± 3.4	4.4 ± 2.7	5.8 ± 4.2	3.1 -20.5	*0.5209*
Deficiency of					
Folate—no. (%)	6/16 (37.5)	4/8 (50.0)	2/8 (25.0)		*0.3017*
Cobalamin—no. (%)	1/15 (6.7)	1/8 (12.5)	0/7 (0)		*0.3329*
***Hemolysis/ inflammation***					
Haptoglobin—g/L	2.3 ± 1.2	2.0 ± 1.6	2.5 ± 0.2	0.14–2.58	*0.6690*
Lactate dehydrogenase—U/L	268 (234–305)	279 (250–305)	247 (192–316)	< 248	*0.3363*
C-reactive protein—mg/L	57.4 (19–107)	61.8 (19–110)	53 (19–101)	< 5.0	*0.6708*
Serum C3—g/L	1.2 ± 0.3	1.3 ± 0.3	1.2 ± 0.3	0.82–1.93	*0.8194*
Serum C4—g/L	0.3 ± 0.1	0.2 ± 0.1	0.3 ± 0.1	0.15–0.57	*0.1071*
***Serologic ANCA titer***					
PR3-ANCA—IU/mL	–	40.5 (30–101)	0.2 (0.2–0.2)	< 3.5	** < *0.0001***
MPO-ANCA—IU/mL	–	0.2 (0.2–0.3)	57.0 (28–109)	< 2.0	** < *0.0001***
***Kidney injury***					
Peak creatinine—mg/dL	2.9 (1–5)	1.8 (1–3)	4.3 (2–6)	0.7–1.2	***0.0050***
eGFR—mL/min./1.73m^2^	21.4 (10–53)	28.2 (14–80)	14.0 (9–39)	> 60	***0.0147***
BUN—mg/dL	45 (29–76)	41 (29–67)	66.0 (30–92)	9–21	*0.0707*
Potassium—mM	4.3 (4–5)	4.2 (4.1–4.4)	4.3 (4.0–4.9)	3.5–4.6	*0.5144*
AKIN I—no. (%)	5 (9.6)	3 (11.1)	2 (8.0)		*0.7038*
AKIN II—no. (%)	5 (9.6)	4 (14.8)	1 (4.0)		*0.1863*
AKIN III—no. (%)	23 (44.2)	7 (25.9)	16 (64.0)		***0.0057***
***CKD stages***					
G1: ≥ 90 mL/min./1.73m^2^	21 (40.4)	13 (48.2)	8 (32.0)		
G2: 60—89 mL/min./1.73m^2^	21 (40.4)	10 (37.0)	11 (44.0)		
G3: 30—59 mL/min./1.73m^2^	5 (9.6)	3 (11.1)	2 (8.0)		0.3585*
G4: 15—29 mL/min./1.73m^2^	3 (5.8)	0 (0)	3 (12.0)		0.2326**
G5: < 15 mL/min./1.73m^2^	2 (3.8)	1 (3.7)	1 (4.0)		0.1709***
***Disease course***					
Newly diagnosed—no. (%)	39 (75.0)	20 (74.1)	19 (76.0)		*0.8727*
Relapsed—no. (%)	9 (17.3)	5 (18.5)	4 (16.0)		*0.8104*
***Treatment at time of rbx***					
RTX—no. (%)	4 (7.7)	1 (3.7)	3 (12.0)		
CYC—no. (%)	5 (9.6)	1 (3.7)	4 (16.0)		
PEX—no. (%)	11 (21.2)	6 (22.2)	5 (20.0)		
KRT—no. (%)	13 (25.0)	5 (18.5)	8 (32.0)		
Steroid pulse—no. (%)	34 (65.4)	16 (59.3)	18 (72.0)		0.638****
***Forms of anemia***					
Anemic—no. (%)	43/52 (82.7)	22/27 (81.5)	21/25 (84.0)		*0.8104*
ACD—no. (%)	8/28 (28.6)	3/12 (25.0)	5/16 (31.3)		*0.7171*
Due to deficiency of Iron—no. (%)	8/28 (28.6)	3/12 (25.0)	5/16 (31.3)		*0.7171*
Folate—no. (%)	6/16 (37.5)	4/8 (50.0)	2/8 (25.0)		*0.3017*
Cobalamin—no. (%)	1/15 (6.7)	1/8 (12.5)	0/7 (0)		*0.3329*
Renal—no. (%)	9/52 (17.3)	3/27 (11.1)	6/25 (24.0)		*0.2196*
***Glomerular lesions***					
Normal—% of total	0.49 ± 0.29	0.58 ± 0.24	0.39 ± 0.3		***0.0120***
Necrosis—% of total	0.15 (0.0–0.45)	0.14 (0.0–0.4)	0.15 (0.0–0.48)		*0.8112*
Crescents—% of total	0.31 (0.1–0.6)	0.28 (0.1–0.5)	) 0.35 (0.1–0.7)		*0.1906*
Sclerosis—% of total	0.05 (0.0–0.3)	0.07 (0.0–0.18)	0.08 (0.0–0.4)		*0.3821*
***Prevalence of anemia by Berden’s classification***					
Crescentic—no. (%)	17/17 (100)	7/7 (100)	10/10 (100)		*–*
Focal—no. (%)	18/25 (72.0)	13/16 (81.3)	5/9 (55.6)		*0.1696*
Mixed—no. (%)	6/7 (85.7)	3/4 (75.0)	3/3 (100)		*0.3496*
Sclerotic—no. (%)	3/3 (100)	0/0 (0)	3/3 (100)		*–*
***Bowman’s capsule rupture***					
Focal—% of total (90%ile)	0.83	0.83	0.93		*0.3147*
Diffuse—% of total (90%ile)	0.18	0.45	0.15		*0.7364*
***Lesions analogous to Banff***					
*i* (0/1/2/3/x)—score	40/9/0/0/3	22/3/0/0/2	19/6/0/0/0		*0.2695*
*t* (0/1/2/3/x)—score	22/21/3/3/3	13/9/1/2/2	9/12/3/1/0		*0.8981*
*v* (0/1/2/3/x)—score	31/0/6/5/10	20/1/0/2/4	12/5/0/3/5		*0.0963*
*g* (0/1/2/3/x)—score	10/5/29/5/2	3/4/16/2/2	8/1/13/3/0		*0.2050*
*ci* (0/1/2/3/x)—score	10/20/19/4/	7/11/9/0/0	2/10/10/4/1		*0.0764*
*ct* (0/1/2/3/x)—score	4/30/12/3/3	4/17/4/0/2	0/14/8/3/0		***0.0347***
*ah* (0/1/2/3/x)—score	31/11/4/2/4	18/4/3/0/2	14/7/1/2/1		*0.2309*
*ptc* (0/1/2/3/x)—score	45/5/0/0/2	23/2/0/0/2	22/3/0/0/0		*0.6374*
*ti* (0/1/2/3/x)—score	17/23/8/1/3	14/8/2/1/2	4/15/6/0/0		***0.0136***
*i-IFTA* (0/1/2/3/x)—score	8/11/11/19/3	6/5/5/9/2	2/6/7/10/0		*0.4795*
*t-IFTA* (0/1/2/3/x)—score	16/32/1/0/3	10/15/0/0/2	6/18/1/0/0		*0.3210*

Continuous variables were tested for normal distribution utilizing the Shapiro–Wilk test. Normally distributed continuous variables are presented as mean ± SD, all non-normally distributed continuous variables are itemized as median (IQR), except for Bowman’s capsule rupture, whose variables are described as 90% percentile. Group comparisons were performed between two subgroups PR3-ANCA and MPO-ANCA by means of unpaired student’s t-test (normal distribution) or Mann–Whitney test (non-normal distribution). Nonparametric between-group comparisons were performed with Chi-Square test. Comparison of CKD stages: (*), G1-5, (**), G3-5, (***), G4-5

Abbreviations: *ACD* anemia of chronic disease, *ah* arteriolar hyalinosis, *AKIN* acute kidney injury network, *ANCA* anti-neutrophil cytoplasmic antibody, *BMI* body mass index, *BP* blood pressure, *BUN* blood urea nitrogen, *BVAS* Birmingham Vasculitis Activity Score, *bx* biopsy, *C3* complement factor 3, *C4* complement factor 4, *ci* interstitial fibrosis, *CKD* chronic kidney disease, *ct* tubular atrophy, *CYC* cyclophosphamide, *eGFR* estimated glomerular filtration rate, *g* glomerulitis, *GFR* glomerular filtration rate, *i* interstitial inflammation, *IFTA* interstitial fibrosis/ tubular atrophy, *i-IFTA* inflammation in areas of IFTA, *IQR* interquartile range, *KRT* kidney replacement therapy, *MCH* mean corpuscular hemoglobin, *MCHC* mean corpuscular hemoglobin concentration, *MCV* mean corpuscular volume, *MPO* myeloperoxidase, *no.* number, *PEX* plasma exchange, *PR3* proteinase 3, *ptc* peritubular capillaritis, *RBC* red blood cells, *rbx* renal biopsy, *RTX* rituximab, *t* tubulitis, *ti* total inflammation, *t-IFTA* tubulitis in areas of IFTA, *v* intimal arteritis, x excluded/not available, *90%ile* 90% percentile, ****, comparison of all treatment measures

### Diffuse rupture of Bowman’s capsule and peritubular capillaritis are associated with low hemoglobin levels in ANCA-associated renal vasculitis

**Fig. 2:**
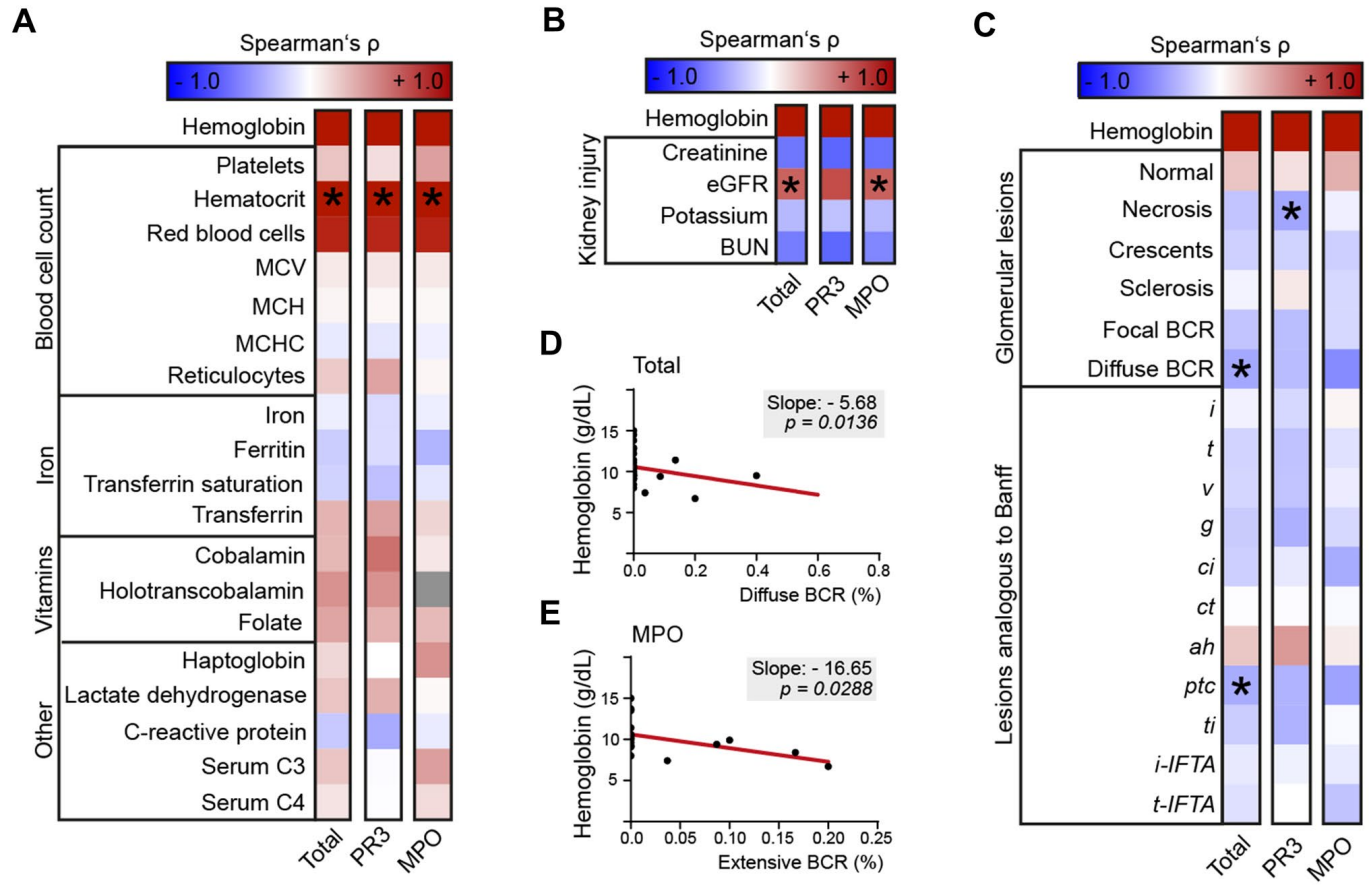
Diffuse rupture of the Bowman’s capsule and peritubular capillaritis are associated with anemia in ANCA-associated renal vasculitis. **A**–**C** Clinicopathological analyses of serum hemoglobin levels with laboratory parameters, and histopathological items in the total ANCA cohort, PR3-ANCA and MPO-ANCA. Heatmaps visualize mean values of Spearman’s ρ, while asterisks represent *p* < *0.05* in the multivariable linear regression analysis including variables of one predefined cluster as indicated by black rectangles. Grey boxes within each heatmap reflect no analysis due to data absence of the respective parameters. **D**, **E** Simple linear regressions between serum hemoglobin levels and diffuse BCR. Slope is indicated by red line. Abbreviations: *ah* arteriolar hyalinosis, *ANCA* anti-neutrophil cytoplasmic antibody, *BCR* Bowman’s capsule rupture, *BUN* blood urea nitrogen, *C3* complement factor 3, *C4* complement factor 4, *ci* interstitial fibrosis, *ct* tubular atrophy, *eGFR* estimated glomerular filtration rate, *g* glomerulitis, *i* interstitial inflammation, *i-IFTA* inflammation in areas of interstitial fibrosis and tubular atrophy, *MCH* mean corpuscular hemoglobin, *MCHC* mean corpuscular hemoglobin concentration, *MCV* mean corpuscular volume, *MPO* myeloperoxidase, *PR3* proteinase 3, *ptc* peritubular capillaritis, *t* tubulitis, *ti* total inflammation, *t-IFTA* tubulitis in areas of interstitial fibrosis and tubular atrophy, *v* intimal arteritis

Next, we analyzed correlations between hemoglobin levels and clinicopathological parameters in the total cohort, and separately in MPO-ANCA and PR3-ANCA renal vasculitis. Regarding laboratory anemia parameters, no significant associations with serum hemoglobin levels were observed (Fig. 2A). Serum hemoglobin levels were associated with eGFR in the total cohort (*p* < 0.0005, *r* = 0.695, Fig. 2B; *β* = 0.539, *p* < 0.001, Table 2), and the MPO-ANCA subgroup (*p* < 0.0005, *r* = 0.699, Fig. 2B; *β* = 0.679, *p* = 0.008, Table 2).

**Table 2:** Stepwise multivariable linear regressions with hemoglobin levels as dependent variable

	*β*	*p* value
***Red blood cells***		
Platelets (MPO)	− 0.04	*0.217*
Hematocrit (Total/PR3/MPO)	0.993/1.027/1.002	** < *0.001/* < *0.001/* < *0.001***
Red blood cells (Total/PR3/MPO)	0.015/− 0.038/0.007	*0.794/0.64/0.947*
***Kidney injury***		
Peak creatinine (Total/PR3/MPO)	0.214/− 0.002/0.180	*0.33/0.995/0.539*
eGFR (Total/PR3/MPO)	0.539/0.488/0.679	** < *0.001/****0.09/****0.008***
Potassium (Total)	− 0.141	*0.175*
BUN (Total/PR3/MPO)	− 0.116/− 0.205/− 0.051	*0.529/0.230/0.735*
***Glomerular lesions***		
Fraction of normal glomeruli (Total)	0.233	*0.135*
Fraction of necrotic glomeruli (Total/PR3)	− 0.175/− 0.424	*0.258/****0.028***
Diffuse BCR (Total/MPO)	− 0.374/− 0.402	***0.014/****0.079*
***Lesions analogous to Banff***		
Peritubular capillaritis (Total/MPO)	− 0.358/− 0.279	***0.013/****0.181*
Interstitial fibrosis (MPO)	− 0.304	*0.147*
Arteriolar hyalinosis (PR3)	0.387	*0.061*

Independent statistical evaluation of variables offering a Spearman's ρ more than ± 0.4 in the correlation matrix by means of stepwise multivariable linear regression. Per analysis variables of the same cluster (indicated by italic headings) and subgroup (indicated by group name in brackets) were included

Abbreviations: *ANCA* anti-neutrophil cytoplasmic antibody, *BCR* Bowman’s capsule rupture, *BUN* blood urea nitrogen, *eGFR* estimated glomerular filtration rate, *MPO* myeloperoxidase, *p value* probability value, *PR3* proteinase 3, *β* beta coefficient

Among tubulointerstitial lesions, decreased serum hemoglobin levels correlated with mild peritubular capillaritis in the total cohort (*p* = 0.005, *r* = − 0.399, Fig. 2C; *β* = − 0.358, *p* = 0.013, Table 2; * p* = 0.0126, 1/slope = − 18.07), which was slightly hinted in MPO-ANCA-positive renal vasculitis (*p* = 0.029, *r* = − 0.446, Fig. 2C), but did not reach statistical significance in the multivariable and simple linear regression analyses (*β* = − 0.279, *p* = 0.181, Table 2; *p* = 0.0752, 1/slope = − 16.11).

Regarding glomerular lesions, necrotic glomeruli were significantly associated with low serum hemoglobin levels in PR3-ANCA (*p* = 0.025, *r* = − 0.430, Fig. 2C; *β* = − 0.424, *p* = 0.028, Table 2). In the total cohort, a significant correlation between decreased serum hemoglobin levels and the occurrence of diffuse BCR was identified (*p* = 0.005, *r* = − 0.423, Fig. 2C; *β* = − 0.374, *p* = 0.014, Table 2), which was further tested by simple linear regression featuring statistical significance (*p* = 0.0136, slope = − 5.68, Fig. 2D) and was hinted in the MPO-ANCA cohort (*p* = 0.013, *r* = − 0.546, Fig. 2C; *p* = 0.0288, slope = − 16.65, Fig. 2F).

## Discussion

The aim of this study was to identify histopathological correlates of low hemoglobin levels in ANCA-associated renal vasculitis. First, we described the prevalence of anemia in our patient cohort, which accounted for 82.7% and we further observed a prevalence of only 17.3% for renal anemia. Given the prevalence of cobalamin and folate deficiency, we observed a minor impact on our patient cohort. We systematically described the characteristics of our patient cohort, which included a high prevalence of severe AKI occurring in up to 64% (in the MPO-ANCA subgroup), and revealed newly diagnosed disease in 75% of cases. Moreover, the application of immunosuppressive treatment measures at, or shortly before, renal biopsy was equally distributed among the subgroups and, besides a frequently applied steroid pulse, RTX and CYC were only given in less than 10% of the total cohort, while PEX or KRT were performed in no more than 25%. We performed correlative analyses regarding histopathological features and hemoglobin levels at the time of renal biopsy regardless of current anemia classification. In line with recent studies, we identified low serum hemoglobin levels correlated with decreased kidney function as assessed by eGFR reduction in the total cohort and in the MPO-ANCA subgroup [2, 4]. Even though kidney function was more severely decreased in MPO-ANCA renal vasculitis, distribution of CKD stages was not significantly different between subgroups implying that acute renal damage patterns, which were observed mostly in the MPO-ANCA subgroup, might contribute to the association of eGFR reduction and low hemoglobin levels. Interestingly, we found no associations between low hemoglobin levels and the histopathological items that were most severely affected in the MPO-ANCA subgroup, such as Banff-scored tubular atrophy and a decreased fraction of normal glomeruli. The features that are generally assumed to functionally compound decreased kidney function as histopathological correlates, such as interstitial fibrosis, were also not correlated with low hemoglobin levels. Of note, we analyzed the prevalence of anemia as assessed by Berden’s classes and found that all patients with crescentic class also had hemoglobin levels in the anemic range.

Next, we identified a correlation of peritubular capillaritis and low hemoglobin levels reflecting anemia severity. Erythropoietin production is oxygen-regulated and dependent on hypoxia-inducible factor 2 (HIF-2), wherein peritubular renal interstitial fibroblast-like cells and pericytes are mainly contributory [6]. During kidney injury, these cells transdifferentiate into myofibroblasts, thus, the capability of EPO production is forfeited resulting in a diminished stimulation of erythropoiesis in the bone marrow (reflected by a reduced reticulocyte production index), finally leading to anemia [35]. Our finding of peritubular capillaritis correlating with anemia severity aligns with this pathophysiological comprehension of anemia causes. From research on kidney transplantation, it is known that development of interstitial injury is strongly influenced by microvascular injury of the peritubular capillaries [12], which could reflect one compound of tubulointerstitial impairment promoting a relative EPO deficiency. Independent occurrence of peritubular capillaritis together with tubulointerstitial inflammation without glomerulonephritis was described in some case reports [14–16], which furthermore points out that vascular injury impairs the tubulointerstitial compartment, hence it might affect EPO-producing cells. On the other hand, hypoxic conditions accompanying anemia promote microvascular inflammation, which might explain why we only observed mild injury of the peritubular capillary network. Moreover, we identified diffuse BCR as being associated with low hemoglobin levels in ANCA-associated renal vasculitis. We recently reported on the concept of BCR enabling a connection of the glomerular and tubulointerstitial space, which further promotes tubulointerstitial inflammation and immune cell infiltration due to loss of an immunological barrier, whose integrity is sustained by Bowman’s capsule under physiological conditions [17, 18]. Hence, periglomerular EPO-producing cells might be affected, additionally contributing to a relative EPO deficiency. Furthermore, an association between diffuse BCR and anemia severity was suggested in the MPO-ANCA subgroup, while glomerular necrosis and low hemoglobin levels correlated in the PR3-ANCA subgroup implying distinct modes of hypoxia-induced kidney injury in different subtypes of ANCA-associated renal vasculitis.

We provide insights into histopathological correlates that potentially influence the clinical occurrence of anemia in ANCA-associated renal vasculitis. We think that the identified correlations of low hemoglobin levels and vascular injury represented by diffuse BCR and peritubular capillaritis reflect an additional aspect of anemia pathophysiology in ANCA-associated renal vasculitis, apart from the current understanding of CKD-associated anemia. On the other hand, the identified correlations might represent a special hypoxic susceptibility of intrarenal lesions, such as BCR and peritubular capillaritis, in ANCA-associated renal vasculitis.

The main limitations of this study include the small patient cohort, the single center experience and the retrospective design, the clinical characterization of the patients’ background and the limited cases of diffuse BCR and peritubular capillaritis. As a future study, investigation of histopathological correlates in association with hemoglobin levels in a larger patient cohort with a multi-center study design would be of interest. Moreover, assessment of serum EPO levels for diagnosis of renal anemia poses a supplementary aspect of future studies, although not recommended to be routinely measured for the diagnosis or management of anemia for patients with CKD [29].

## Conclusions

To the best of our knowledge, this is the first report of specific histopathological lesions associated with anemia severity in ANCA-associated renal vasculitis. The finding that peritubular capillaritis and Bowman’s capsule rupture correlate with low hemoglobin levels may add to the notion that histopathological lesions might link inflammatory vascular injury to relative EPO deficiency and renal anemia in ANCA-associated renal vasculitis.

## Data Availability

Deidentified data are available on reasonable request from the corresponding author.
